# Strong link between Earth’s oxygen level and geomagnetic dipole revealed since the last 540 million years

**DOI:** 10.1126/sciadv.adu8826

**Published:** 2025-06-13

**Authors:** Weijia Kuang, Ravi Kopparapu, Joshua Krissansen-Totton, Benjamin J. W. Mills

**Affiliations:** ^1^ Geodesy and Geophysics Laboratory, NASA Goddard Space Flight Center, Greenbelt, MD 20771, USA.; ^2^ Sellers Exoplanet Environments Collaboration (SEEC), NASA Goddard Space Flight Center, Greenbelt, MD 20771, USA.; ^3^ Planetary Environments Laboratory, NASA Goddard Space Flight Center, Greenbelt, MD 20771, USA.; ^4^ Department of Earth and Space Sciences/Astrobiology Program, University of Washington, Seattle, WA, USA.; ^5^ School of Earth and Environment, University of Leeds, Leeds LS2 9JT, UK.

## Abstract

Earth is the only known rocky planet to support complex life forms that use oxygen and to have a strong intrinsic magnetic field in much of its history, prompting speculation that Earth’s magnetic field and habitability are related on geological timescales. We search for possible observational evidence for such a relationship by examining evolutions of the virtual geomagnetic axial dipole moment and the atmospheric oxygen level over the past 540 million years. We find that both exhibit strong linearly increasing trends, coupled with a large surge in magnitude between 330 and 220 million years ago. Our time series analysis and statistical tests show that both are highly correlated, with the maximum correlation reached when there is no time lag between the two. Our findings suggest unexpected strong connections between the geophysical processes in Earth’s deep interior, the surface redox budget, and biogeochemical cycling.

## Teaser

Atmospheric oxygen level evolves in tandem with geomagnetic dipole moment since early Cambrian.

## INTRODUCTION

Earth is the only known terrestrial planet to have life, and the earliest unambiguous evidence of life on Earth, in the form of microorganisms, is 3.5 billion years (Gyr) old (*1*) and potentially much older (*2*, *3*). How could Earth maintain its habitat through numerous extreme internal geological events and external storms from space over geological timescales? This has become one of the fundamental science questions in understanding evolution of life and searching for habitable worlds. The search for the answers includes a recent focus on whether the presence of Earth’s strong intrinsic magnetic field (called the geomagnetic field herein) could be a necessary condition of Earth’s habitability because the geomagnetic field has also been present throughout much of Earth’s history, as shown from paleomagnetic records with the ages comparable to those of life on Earth (*4*, *5*). Analysis of space weather shows that the geomagnetic field can prevent or reduce Earth’s atmospheric escape and erosion due to, e.g., ionization and ohmic heating arising from solar winds and solar energetic particles (SEPs) from coronal mass ejections (CMEs) (*6*–*9*), and can protect life on Earth’s surface from x-ray and extreme ultraviolet (XUV) radiation. However, opposing results may also occur, depending on the field properties and solar activities (*10*, *11*). Regardless, possible consequences of the geomagnetic field for Earth’s surface environment will continue to be intensively examined, aiming at understanding life on Earth, and at searching for habitable worlds (*12*, *13*).

**Fig. 1.**
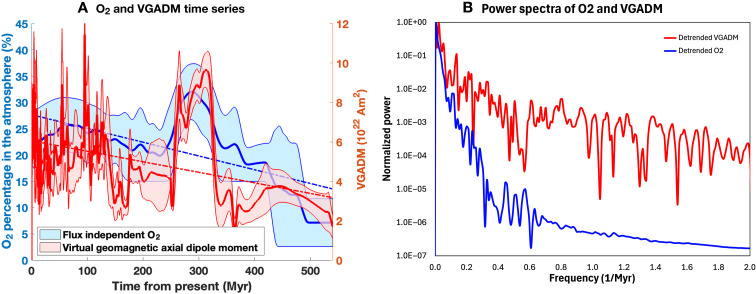
Time variations of the oxygen (O_2_) content and the VGADM in the past 540 million years. (**A**) Time series of O_2_ (blue) and VGADM (red). The solid lines are the mean values, and the banded regions are the data uncertainties. The dashed lines are linear trends. (**B**) Scaled power spectra of the detrended mean O_2_ and the detrended mean VGADM. The spectra are scaled by their corresponding strongest mode.

Several studies have provided important snapshots of the geomagnetic shielding of atmospheric escape and of XUV radiation (*6*–*8*) over time by using various solar activities and geomagnetic properties in simulations. The resultant atmospheric erosion or loss to space can be substantial over geological timescales. For example, the simplified scaling law (*8*) of the oxygen loss due to the extreme XUV fluxes that typically occur around M-dwarfs suggests that the loss of oxygen for an Earth-like planet around such an M-dwarf could reach ~10^18^ kg over 25 million years (Myr), which is comparable to the oxygen mass in the modern Earth’s atmosphere. However, considering the complex evolution of the geomagnetic field (see Fig. 1), Earth’s atmospheric properties and solar fluxes, simple extrapolations from these snapshots may skew our understanding of geomagnetic impacts over geological timescales. On the other hand, research on Earth’s oxygenation history and supercontinent cycles (*14*, *15*) could also link Earth’s oxygen content and the geomagnetic field over geological timescales because the supercontinent cycles—including weathering and degassing processes that regulate surface oxygen (*16*)—alter the thermal heterogeneity across the core-mantle boundary (*17*) and thus affect the geodynamo process in Earth’s fluid core (*18*) over several hundred million years. However, current long-term Earth system models do not include the geomagnetic field and have not explored these potential links between atmospheric oxygen and interior processes.

Thus, we take a very different approach by testing whether observational evidence supports a link between the evolution of the geomagnetic field and of Earth’s habitable environment. Two completely independent datasets are available for this purpose, one is the virtual geomagnetic axial dipole moment (VGADM) derived from paleomagnetic records, and the other is the atmospheric oxygen level derived from various geochemical proxies. Both cover the period over the past half billion years. Their correlation properties over the period may provide the first clue about potential consequences of the geomagnetic field for Earth’s habitat.

One of the most important events in the evolutionary history of Earth is the emergence of oxygenic photosynthesis. This mechanism caused oxygen to be the second most dominant gas in Earth’s atmosphere, after nitrogen. Several geochemical proxy records (*19*, *20*) have mapped out the long-term evolution of oxygen partial pressure over the last ~3 Gyr. These proxies indicate that Earth’s atmospheric oxygen levels started out as low as ~0.1 parts per trillion [or 10^−10^ PAL (present atmospheric level) of 21% by volume] and intermittently went up to as high as ~35% (*21*). Notable in this evolutionary history is the “Great Oxidation Event” (GOE) that occurred between 2.4 and 2.2 billion years ago (Ga), when the planet transitioned to an oxygenated atmosphere (*22*). Although the cause of the oxygenation event is debated (*23*, *24*), further oxygenation events occurred post-GOE in the Neoproterozoic (~1 to 0.54 Ga) (*25*), raising the O_2_ level toward the present Earth-level concentrations. Proxies of atmospheric oxygen levels in the Phanerozoic (~540 Myr to present) indicate a linearly increasing level that includes a surge in the late Paleozoic, around 350 to 250 million years ago (Ma), potentially increasing up to as much as ~35% of the atmosphere before decreasing to approximately modern levels. Although the cause of this surge is unclear, a similar linear trend and surge is noticeable in the VGADM data in the same time period (Fig. 1A). Such apparent common trends may have occurred even in the Precambrian; however, we limit our analysis to the Phanerozoic as relatively high time resolution datasets are available for both O_2_ and VGADM, and Precambrian oxygen levels are highly uncertain (*21*).

The origin of the geomagnetic field is well described by the geodynamo theory, which argues that the geomagnetic field is generated and maintained by vigorous convection in Earth’s fluid core, which is driven by thermal and compositional buoyancy released from Earth’s secular cooling and the solid inner core solidification (*26*–*29*). The geodynamo has been active for more than 4 Gyr (*4*, *5*); as such, the dynamo-generated magnetic field intensity and morphology, including the polarity, vary chaotically throughout Earth’s history (*30*, *31*) and are recorded in the magnetic minerals at the time of their formation via several mechanisms (*32*). The records, called the paleomagnetic records, are used to obtain continuous VGADM time series, which is a good proxy for the time-varying geomagnetic field on Earth’s surface. VGADM can be estimated based on paleointensity records, sample geolocations, and the approximation that the geomagnetic field is mainly a dipolar field (*31*). Obviously, the qualities of VGADM, including its temporal resolutions, are controlled by the available samples collected over time.

## RESULTS

**Fig. 2.**
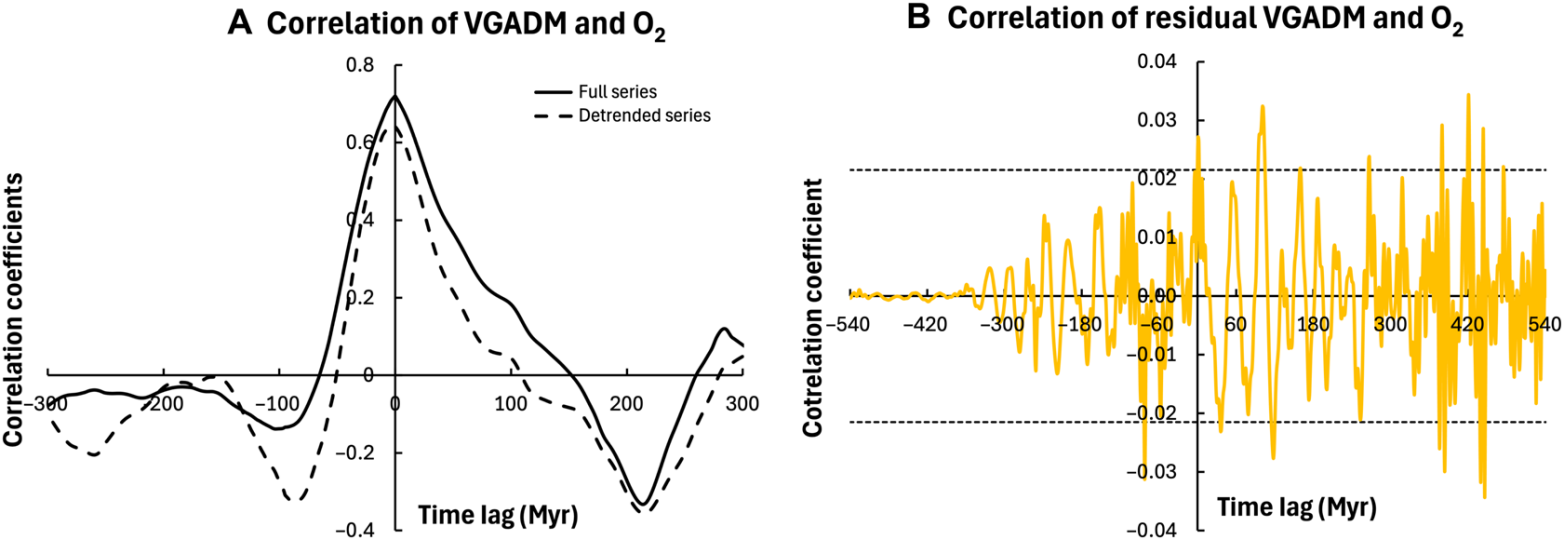
Correlation of VGADM and O_2_ with the 10-Myr band-pass filter. (**A**) Correlogram of the full VGADM and O_2_ series (solid) and the detrended VGADM and O_2_ time series (dashed). (**B**) Correlogram of the residual (the difference between the full and filtered series) VGADM and O_2_. The dotted lines are the significance range ± 2/$\sqrt{N}$ ; values outside the dotted lines are significantly different from zero. This further confirms that VGADM and O_2_ would correlate well only on very long timescales.

The first common feature is the linearly increasing trends in the full VGADM and O_2_ content over the past 540 Myr (the dashed lines in Fig. 1A). This linear trend, called the secular trend here, provides a strong correlation on the longest timescales of the two series. As shown in Fig. 2, the two are strongly correlated, with the maximum correlation coefficient value of 0.72 reached at k=0 (no time lag). The detrended VGADM and O_2_ are still strongly correlated, with the maximum correlation coefficient value of 0.644 reached at k=−1 , which implies a short −1-Myr time lag. However, considering the 2-Myr high band-pass filter applied to both time series (see the Materials and Methods), this is indifferent from no time lag. The slightly lower correlation value for the detrended time series is obviously due to removing the linear trends in the full time series.

**Fig. 3.**
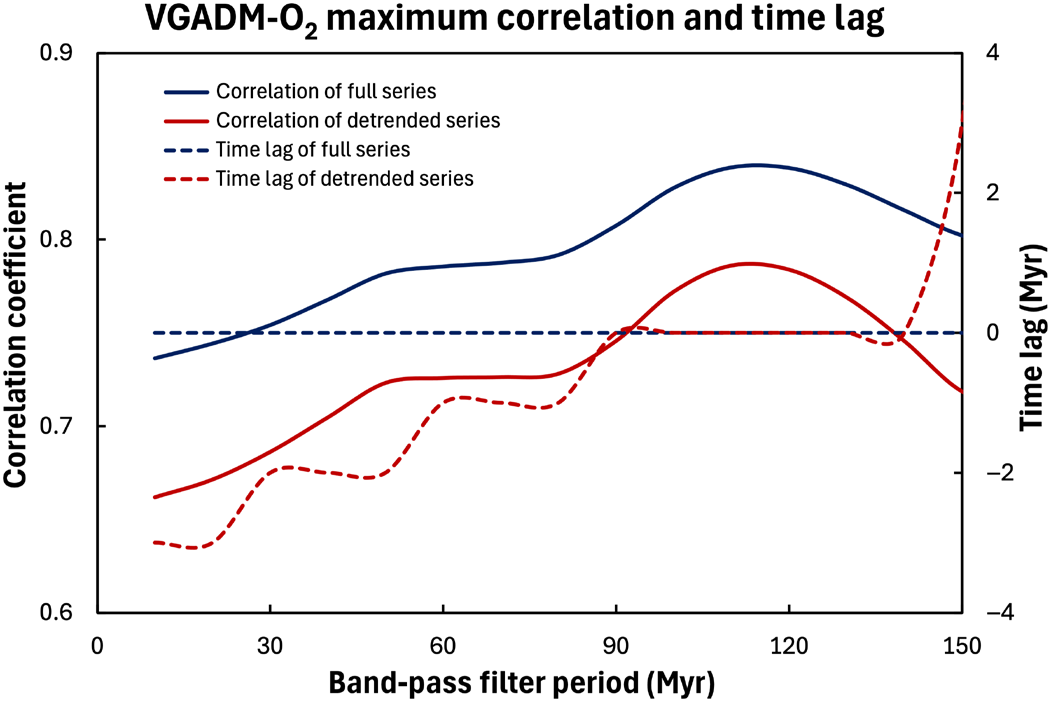
Correlations of VGADM and O_2_ series with different band-pass filters. Maximum correlation coefficients (solid lines) and the corresponding time lags (dashed lines) for the full series (blue) and the detrended (red) series. There is no time lag for the full time series. However, small time lags (less than 4 Myr) appear in the correlation of the detrended time series.

**Fig. 4.**
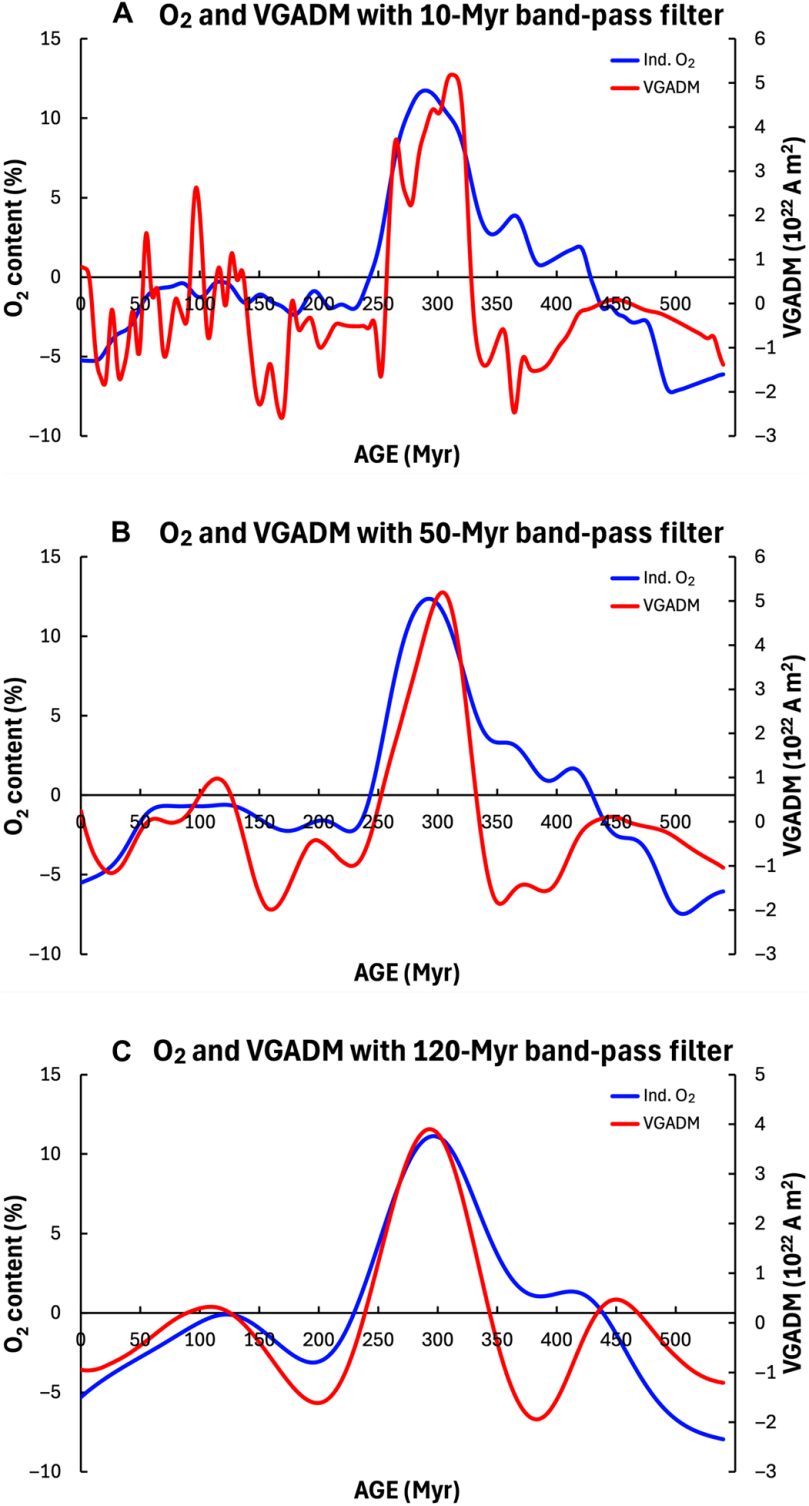
Filtered detrended VGADM and O_2_ series. (**A**) 10-Myr band-pass filter (their correlations are shown in Fig. 2). (**B**) 50-myr band-pass filter. (**C**) 120-Myr band-pass filter. Both VGADM and O_2_ peaked in the 120-Myr time interval between 230 and 350 Myr. However, O_2_ peaked later than VGADM, but the time lag decreases as the band-pass filter period increases, until no lag at all after applying the 120-Myr band-pass filter.

To assess the impacts of high-frequency signals on VGADM and O_2_ correlations, we examined the correlation of the VGADM and O_2_ filtered with a high band-pass filter with the 10-Myr period. As shown in Fig. 2B, their correlation coefficients are very small and mostly insignificant statistically. This is consistent with their power spectra (Fig. 1B), which shows that the relative power of O_2_ is two orders of magnitude weaker than that of VGADM for the periods shorter than 20 Myr and further confirms that VGADM and O_2_ would correlate well only on very long timescales. Next, we evaluate the correlation coefficients of the time series filtered by different band-pass filters. Our findings are summarized in Fig. 3, where the maximum values of the correlograms (see Fig. 2) and the corresponding time lags are shown for both the full and detrended time series of VGADM and O_2_ for different band-pass filter periods. We find that the correlation coefficient increases with the filter periods, implying that VGADM and O_2_ correlation becomes stronger at longer timescales. No time lag is found with the full time series. However, small negative time lags are found for the detrended time series. The time lag decreases as the band-pass filter period increases and vanishes with the periods ranging approximately between 90 and 140 Myr. As shown in Fig. 4, this time lag is mainly because the detrended VGADM and O_2_ peak between 250 and 350 Ma, and O_2_ peaks slightly later than VGADM in time.

**Fig. 5.**
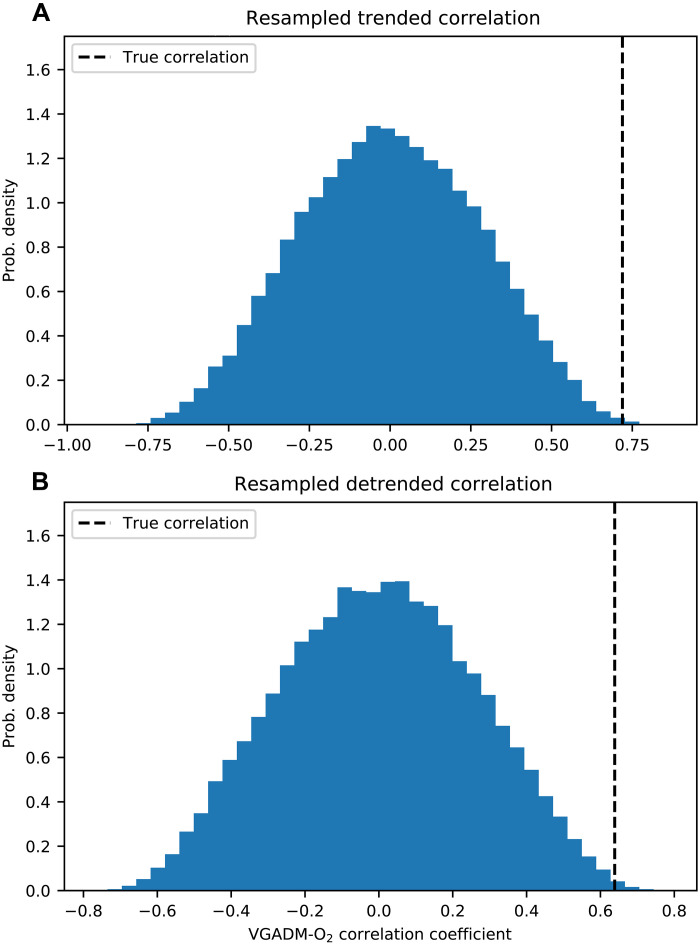
Correlation between oxygen and geomagnetic field proxies is statistically significant. To account for autocorrelation, the oxygen time series was resampled using Fourier series to generate a large number of synthetic time series with the same autocorrelation properties (see the Supplementary Materials). Subplot (**A**) shows the distribution of correlation coefficients when these resampled time series are correlated with VGADM, and subplot (**B**) shows the same calculation when both time series are first detrended then correlated. In both cases, the true correlation coefficient (vertical dashed line) is statistically unlikely compared to the correlation expected by chance (blue distributions).

Considering that both VGADM and O_2_ time series have large uncertainties and are autocorrelated (see Fig. 1), we want to evaluate the significance of our results given the tendency of autocorrelated time series data to correlate by chance, i.e., the effective sample size of autocorrelated data is smaller than the number of samples. Specifically, we adopted a Monte Carlo approach where we generated synthetic O_2_ time series with the same autocorrelation properties as the true proxy data and compared the Monte Carlo distribution of correlations to the correlation coefficient between the two real datasets. The results, which are summarized in Fig. 5, show the same outcome for both the full and the detrended O_2_ data: The true correlation (shown by the vertical dashed line) is statistically unlikely given the level of autocorrelation in the data; the true correlation coefficient is in the 99.9th percentile of the correlation coefficient distribution in both cases. This adds weight to the argument that this correlation is unlikely to be due to chance. In addition, we performed sensitivity tests where we repeated this calculation and resampled the VGADM dataset, included random error in both datasets, and exaggerated the autocorrelation of both datasets—in all three cases, the correlation between O_2_ and geomagnetic field remained statistically significant (see fig. S2 in the Supplementary Materials).

## DISCUSSION

The VGADM and O_2_ data used in our analysis are acquired independently from various sources and are the direct indicators of the geodynamic processes in Earth’s fluid core and the biogeochemical processes on Earth’s surface, respectively. Therefore, the strong correlation between the two may provide the first clue for potential links between the core dynamics, which are responsible for the observed geomagnetic field and the atmospheric redox budget and biospheric processes on Earth’s surface over geological timescales. This could have profound implications for our understanding of Earth’s evolutionary history and, in particular, the evolution of Earth’s surface environment and its relationship with the dynamics in Earth’s interior.

Several possible interpretations may be derived from this long-term correlation. First, the observed correlation could be interpreted to support the conjecture that the strong geomagnetic field is essential in protecting Earth’s atmosphere by reducing oxygen escape because stronger VGADM corresponds to higher O_2_ content in the atmosphere over geological timescales. However, this interpretation is debated given the current understanding of oxygen cycling and nonthermal escape over Earth’s history. The modern oxygen escape flux is ~10^26^ O+ ions/s or 0.005 Tmol O+/year (*33*), whereas the time-averaged O+ polar outflow escape rate over the last 2 to 3 Gyr of Earth history has been estimated not to exceed 0.002 to 0.004 Tmol O+/year (*34*). These fluxes are negligible compared to the oxygen sources and sinks due to magmatic degassing, organic carbon burial, and organic weathering, which are typically ~1 to 10 Tmol O_2_/year (*16*, *35*). Moreover, there are no theoretical reasons to expect the modest observed changes in VGADM over the Phanerozoic to cause O+ escape rates to increase by the required ~2 orders of magnitude to have a substantial influence on atmospheric oxygen evolution, especially because O+ escape rates on Venus (with no dipole field) are less than that of the Earth, around ~2 × 10^24^ to 6 × 10^24^ O+ ions/year (*36*), although one should note that the oxygen level in Venus’ atmosphere is several orders of magnitude lower than that of Earth. In short, nonthermal escape of oxygen is dwarfed by oxygen exchange fluxes between the atmosphere and interior (and the supply of oxygen atoms at Earth’s surface is virtually unlimited). One interesting conjecture is that the near collapse of the paleomagnetic intensity (thus of VGADM) at the end of the Ediacaran may have caused substantial increases in H ion escape and thus oxygenation on the surface over tens of millions of years and longer (*14*). Although the direction of the VGADM-O_2_ correlation is opposite to the conjecture, this study highlights the potential importance of underexplored connections between atmospheric escape and Earth’s magnetic field.

A more plausible scenario is that both O_2_ and VGADM are affected by other geodynamic processes in Earth’s deep interior. As shown in Figs. 1 and 4, the strong correlation between VGADM and O_2_ is mainly due to two outstanding time-varying patterns, the linear increasing trend, and the largest peak in an ~110-Myr period between 220 and 330 Ma. The increasing trend of VGADM starting from the end of Ediacaran and the beginning of Cambrian may be tied to the start of the solid inner core solidification (*14*, *37*). The compositional buoyancy released from the inner core solidification could be much more efficient than the thermal buoyancy previously available to drive vigorous core convection, which, in turn, produces a strong geomagnetic field via the core dynamo process.

However, this process alone may not be sufficient to account for the strong spike in VGADM between 220 and 330 Ma, which coincides with the formation and dissemination of the supercontinent Pangea and the Kiaman Reversed Polarity Superchron. Therefore, it is also possible that supercontinents could play a key role in the long-term evolution of both the geomagnetic field and the atmospheric O_2_ content. For example, both the mean heat flux and its lateral variation across the core-mantle boundary can be strongly affected by the supercontinents. Such heterogeneity could place strong constraints on geodynamo in the core and thus the field intensity and its polarity (*17*, *38*, *39*). In addition, changes in the mantle dynamic processes during the supercontinents could modulate the plausible mechanisms governing the surface redox budget (*40*–*42*) and operate on a sufficiently long timescale that they could do so without introducing an observable lagged response (see the Supplementary Materials). Therefore, more systematic investigation of the geodynamo and, e.g., the crustal recycling for O_2_ production, is needed with the thermal conditions imposed by supercontinents.

Obviously, one could also argue that the correlation between VGADM and O_2_ is purely coincidental and does not provide any important geophysical and geochemical implications because 540 Myr of age accounts only for a small fraction of Earth’s history and because this period includes substantial events in plate tectonics and the inner core formation. However, this scenario could not explain well the similarity between the time variations of VGADM and O_2_ on timescales of 100 Myr and shorter, which is certainly much shorter than the history of the inner core formation, and the supercontinent cycles. However, should higher temporal resolution and longer record paleomagnetic and O_2_ data were available, we would be able to better determine correlations between the two time series in the higher-frequency (sub-million year) domains or improved knowledge of the impacts of supercontinent cycles on the geomagnetic field generation in Earth’s fluid core and the atmospheric oxygen content on the surface.

## MATERIALS AND METHODS

We reassess a recent multiproxy atmospheric O_2_ curve for this study. A previous work has used a combination of different independent proxies to define a likely window for the Phanerozoic atmospheric O_2_ evolution (*21*). These are (i) the abundances of fossilized charcoal in sediments, reflective of the prevalence of wildfire, which is highly sensitive to O_2_ concentration (*43*–*47*); (ii) the abundance of reduced carbon and sulfur compounds in sediments, reflective of the redox balance of the surface system; (iii) carbon isotope offsets, indicative of photosynthetic efficiency, itself dependent on O_2_ levels; (iv) an upper limit for the atmospheric O_2_ defined by the prevalence of a broadly anoxic ocean interior during some periods and the downwelling rate of oxygen required to achieve this; (v) the assessed minimum requirements of the contemporary shallow marine biosphere. We combined these estimates in line with a previous work (*21*), but we omit the additional curve that is based on an inversion of carbon isotope record to estimate oxygen production fluxes (*48*). We omit this proxy estimate because it is dependent on assumed geodynamic fluxes that control CO_2_ inputs and their isotopic compositions, which could create circularity when compared to the paleomagnetic records below.

**Fig. 6.**
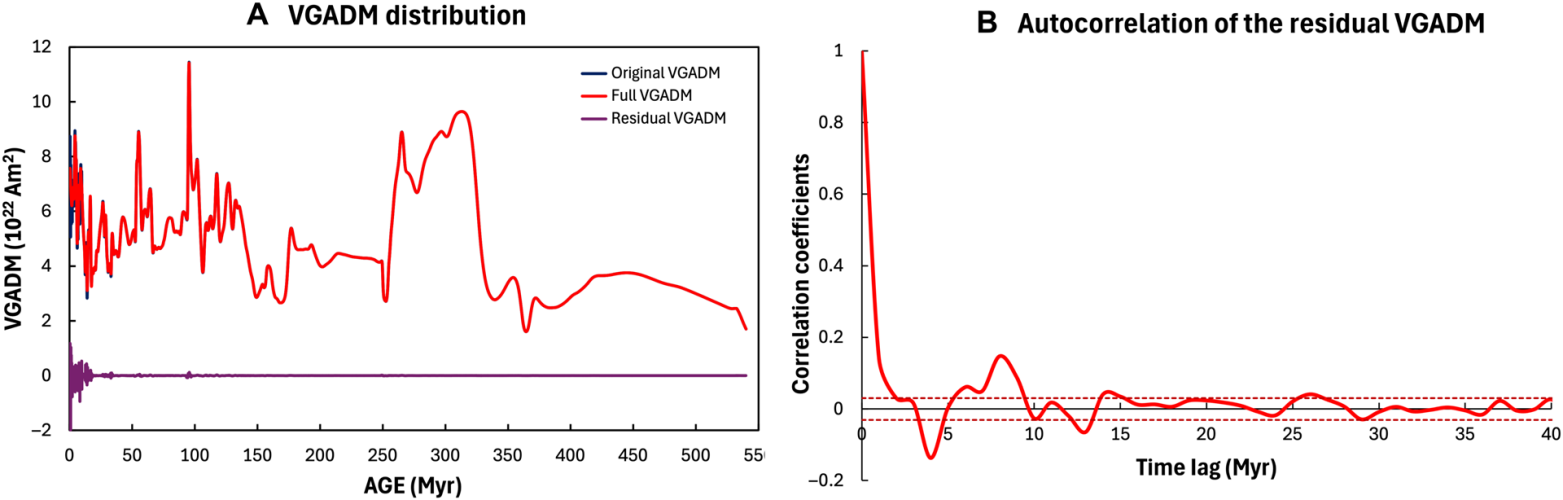
Full VGADM data series for correlation studies. (**A**) Original VGADM (black), the full VGADM (red), and the residual VGADM (purple) removed from the correlation analysis. The residual includes all signals with periods <2 Myr. (**B**) Autocorrelogram of the residual VGADM. The dotted lines are the same as those defined in Fig. 2B.

We use VGADM from the MCADMv1a paleomagnetic model (*31*) in the past 540 Myr when the O_2_ proxy time series are available. Because the detrended O_2_ proxy varies on timescales of 2 Myr and longer (Fig. 1), we first filter the original VGADM time series by removing all signals with periods <2 Myr (see the Supplementary Materials) and then map the values on to the same time grids as the O_2_ dataset. This modified VGADM, which is called the full VGADM time series, is then used for the correlation evaluation. As shown in Fig. 6, the residuals (the difference between the original and the modified VGAD) are negligibly small and do not show long-term autocorrelations. Therefore, it will not affect the correlation results between the modified VGADM and the O_2_ proxy time series. The correlation coefficient *r_k_* (with the subscript *k* denoting the time lag between the two series) follows standard time series analysis methodologies (*49*)

$$r_{k}\left(x,y\right)=\frac{c_{k}\left(x,y\right)}{\sqrt{c_{0}\left(x\right)c_{0}\left(y\right)}},\;c_{k}\left(x,y\right)=\frac{1}{N}\;\sum_{i=1}^{N-k}\left(x_{i}-\underline{x}\right)\left(y_{i}-\underline{y}\right)$$

where *c_k_* is the covariance between the two series.

We focus on finding the maximum correlation coefficients for all possible time lags and for the full time series as well as those filtered with specific low and high period band passes (*50*), so that we can assess on what timescales O_2_ and VGADM correlate the strongest. Given that both VGADM and O_2_ have large uncertainties and temporal autocorrelation, we also use a Monte Carlo statistical approach to test the robustness of the correlation coefficients against large uncertainties in both O_2_ and VGADM datasets.

## Supplementary Materials

### The PDF file includes:

Supplementary Text Figs. S1 to S6 Legends for data S1 and S2 References
