# Supplementary material for: Strong link between Earth’s oxygen level and geomagnetic dipole revealed since the last 540 million years
Source: Sci Adv. 2025 Jun 13;11(24):eadu8826. doi: 10.1126/sciadv.adu8826 (PMC12164983; doi:10.1126/sciadv.adu8826)
Supplement: Supplementary file 1 — Supplementary Text Figs. S1 to S6 Legends for data S1 and S2 References [file sciadv.adu8826_sm.pdf]

Science Advances
AAAS

Supplementary Materials for

Strong link between Earth's oxygen level and geomagnetic dipole revealed since the last 540 million years

Weijia Kuang *et al.*

Corresponding author: Weijia Kuang, weijia.kuang-1@nasa.gov

Sci. Adv. **11**, eadu8826 (2025)
DOI: 10.1126/sciadv.adu8826

The PDF file includes:

Supplementary Text
Figs. S1 to S6
Legends for data S1 and S2
References

Other Supplementary Material for this manuscript includes the following:

Data S1 and S2

1. Bandpass filter

In our correlation analysis, we adopt a high-frequency bandpass filter based on the scheme developed for non-uniform grids (*50*). In this scheme, the filter in the frequency domain is defined as

$$W(\omega) = \{1\ 0 \le |\omega| \le \omega_s, \left(\frac{\pi}{2}\frac{|\omega| - \omega_s}{\delta\omega}\right)\ \omega_s \le |\omega| \le \omega_f, 0\ \omega_f \le |\omega|, \qquad (S1)$$

where $\delta\omega \equiv \omega_f - \omega_s$ is the frequency transition domain of (S1), and the mean $\omega_0 \equiv (\omega_f + \omega_s)/2$ is called the bandpass filter frequency in this paper. Its equivalent form in the time domain is

$$w(t) = \frac{\pi}{2}\frac{sin\ sin\ \omega_f t\ \ +sin\ sin\ \omega_s t}{t[\pi^2 - t^2(\delta\omega)^2]}. \qquad (S2)$$

Obviously, *w*(-*t*) = *w*(*t*). In our analysis, we specify the period *T* first, and the frequency is then given by $\omega = 2\pi/T$. Given a time series $\{i = 1,2,\cdots,N\}$, its filtered series is then of the form

$$\underline{f}(t) = \frac{\sum_{|s_i| \le s_x}\ \ f(t_i)w(s_i)\sigma_i}{\sum_{|s_i| \le s_x}\ \ w(s_i)\sigma_i}, \qquad (S3)$$

where

$$s_i \equiv t - t_i, \qquad \sigma_i \equiv \frac{1}{2}(d_{i-1} + d_i), \qquad d_i \equiv t_{i+1} - t_i, \qquad (S4)$$

and s_x is the upper bound of the summation in (S3). In our analysis, for a given period $T_0 = 2\pi/\omega_0$, the range for the filter (S1) is defined as

$$\omega_f = \frac{2\pi}{T_0 - 3\delta}, \qquad \omega_s = \frac{2\pi}{T_0 + 3\delta}, \qquad (S5)$$

where δ is the grid size of the time series. Unless otherwise specified, the grid size of the time series is $\delta = 1$ myr for all data series.

We first filtered the original MCADMv1a time series by Bonon et al (*31*) with the setting $T_0 = 10^6$ years (and the grid size is $\delta = 50{,}000$ years), and then mapped the filtered time series on the time grid (of 1 myr time step grid up to 540 ma) of the O_2 data. The filtered series and its residual are shown in Fig. 6 of the main text. This time series, called the original VGADM time series, is the dipole moment data used for the correlation analysis with different O_2 time series in this study. In our study, the VGADM and O_2 time series are then filtered with a series of the periods $T_0 = 10\ (i + 1)$, for *i* = 0,14.

2. Monte Carlo resampling to assess statistical significance of correlations

To evaluate the significance of our results given the tendency of autocorrelated time-series data to correlate by chance, we generated synthetic O_2 time-series with the same autocorrelation properties as the true proxy data and compared the Monte Carlo distribution of correlations to the correlation coefficient between the two real datasets. Synthetic, resampled O_2 time series were generated by fitting the O_2 data with a Fourier series, and then resampling the phases for each term. Fig. S1 shows examples of such resampled, synthetic O_2 time-series alongside the true (unmodified) O_2 reconstruction. We retained 32 terms in the Fourier series in nominal calculations, but results are insensitive to the number of terms used to fit and resample the data. The resulting synthetic sample of O_2 time series were then correlated with the magnetic field proxy to generate Fig. 5 in the main text. Calculations were repeated for both trended and detrended time series data, and in each case the true correlation coefficient is in the 99.9th percentile of the correlation coefficient distribution, suggesting the correlation is unlikely due to chance.

We also tested whether results were sensitive to whether only oxygen or both oxygen and paleomagnetic field time series were resampled, as well as sensitivity to uncertainties in each individual time-series. Fig. S2A and fig. S2D are identical to Fig. 5A and Fig. 5B in the main text except that both the O_2 and VGADM time series are fitted with a Fourier series and resampled. Results are virtually the same as the nominal case because the autocorrelation of the O_2 time series dominates the correlation. Fig. S2B and fig. S2E show the results from a different sensitivity test where random error has been added to both O_2 and VGADM time series, either by resampling the reported uncertainties in VGADM or resampling the reported range in O_2 reconstruction. The correlation appears even more significant in this case since the noise-added datasets have less autocorrelation. Finally, fig. S2C and fig. S2F are for the most conservative case where we resampled both time series as a Fourier series with only the first 6 terms, meaning the temporal variance in each dataset is poorly represented and chance autocorrelation is favored. While the significance of the correlation is slightly reduced, the correlation between O_2 and VGADM time series remains statistically significant.

3. Difference between the flux-independent O_2 and the isotope O_2

While we prefer to take O_2 proxies without input from inversion of carbon isotope records, for the reasons to avoid potential correlation due to the flux assumptions which depend on the reconstruction of plate tectonics, in which paleomagnetic records may be utilized (*51*). Here we report additional analysis to compare the flux-independent O_2 proxies and the isotope O_2 proxies and their correlations with VGADM over the past 540

myr. As shown in fig. S3 and fig. S4, the isotope O_2 shows higher temporal variability on time scales shorter than 30 myr. And its maximum correlation with VGADM occurs with a finite time lag around 25 myr (see fig. S5). But both O_2 proxies show strong correlations with VGADM over the past 540 myr.

4. **Exploring lagged responses of O_2 to tectonic forcing.**

It might be expected that O_2, having a multi-million-year residence time in the surface system, should correlate to VGADM with a positive lag time. We show in fig. S6 that lag times are substantially reduced when process changes occur on timeframes far beyond the residence time. We conclude that any lagged response of atmospheric O_2 to tectonic forcing over a supercontinent cycle is sufficiently small to be accounted for in the time error on the atmospheric O_2 proxy record, which is typically at the stage level (~5 myr).

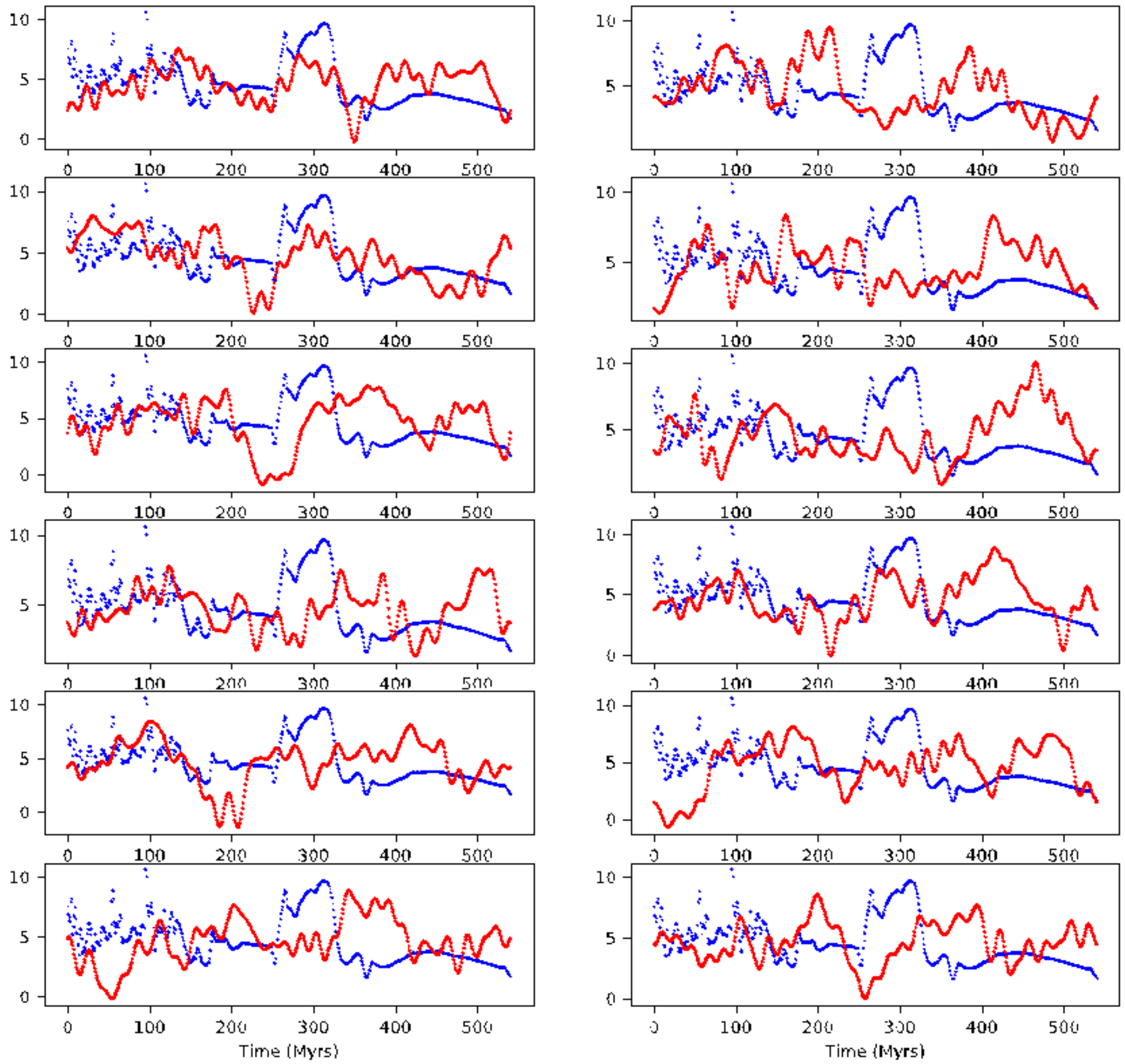

Figure S1. Synthetic time series ensembles for significance test. Examples of synthetic O_2 time-series (red) that are generated from the true O_2 (blue).

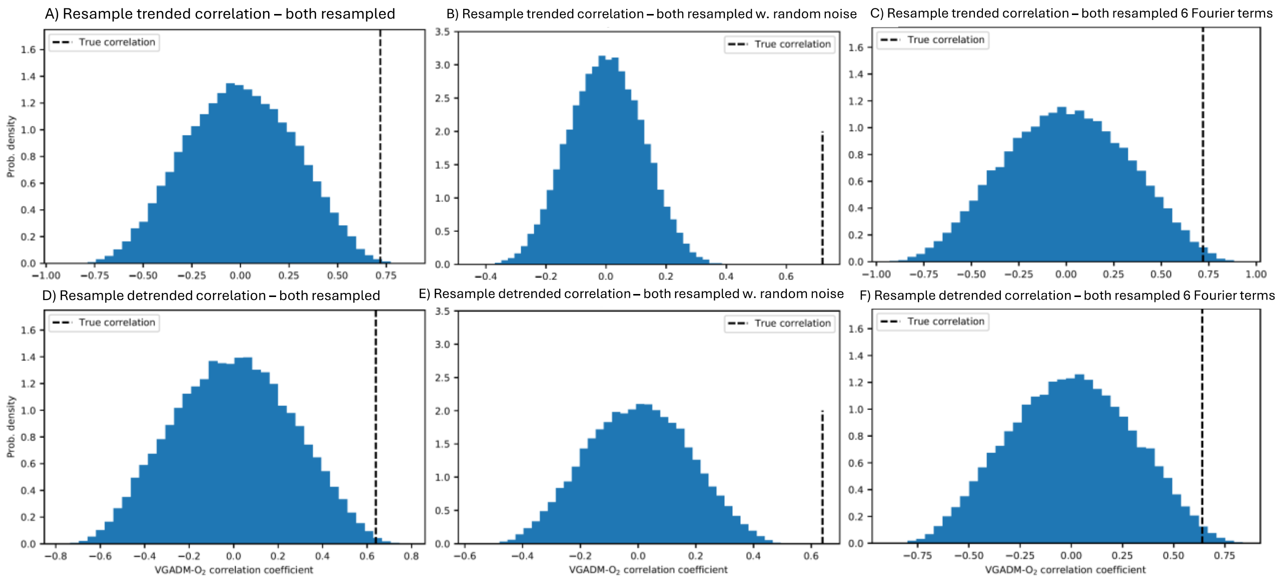

Figure S2. **Correlation coefficients of resembling O_2 and VGADM data.** Statistical significance tests with different scenarios of resampling O_2 and VGADM data.

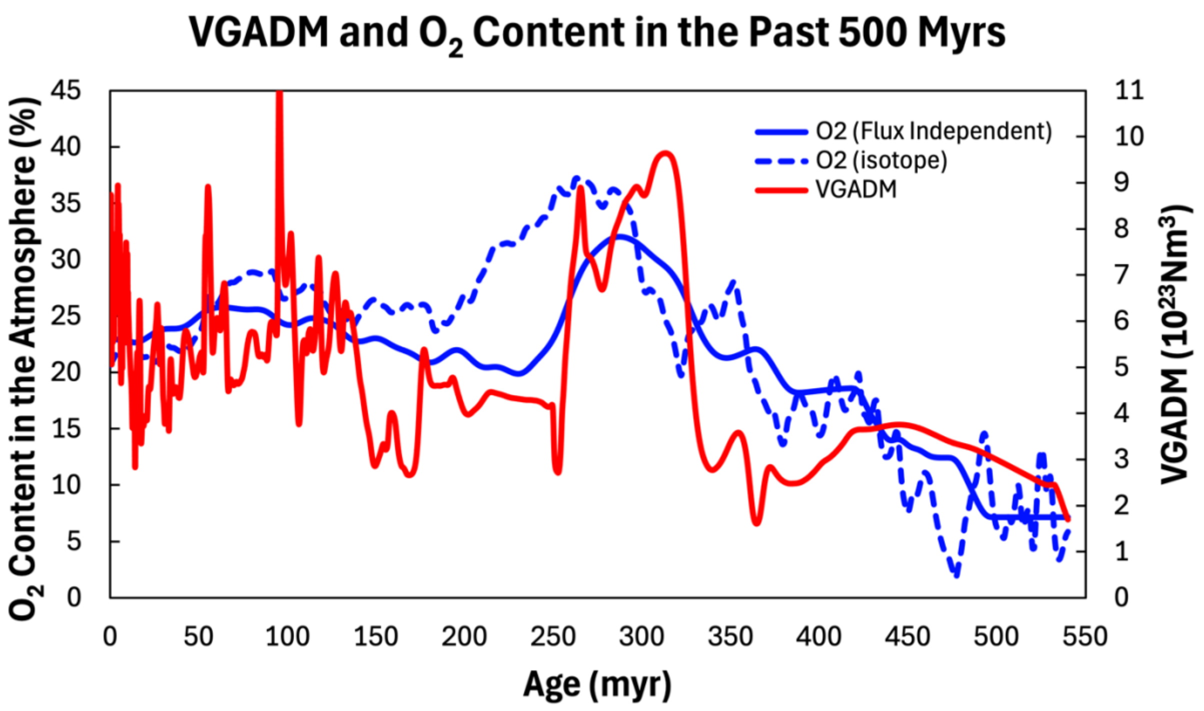

Figure S3. Evolution of VGADM and O_2. This is similar to Fig. 1A in the main text, but with also the O2 proxies from the carbon isotope inversion (blue dashed curve).

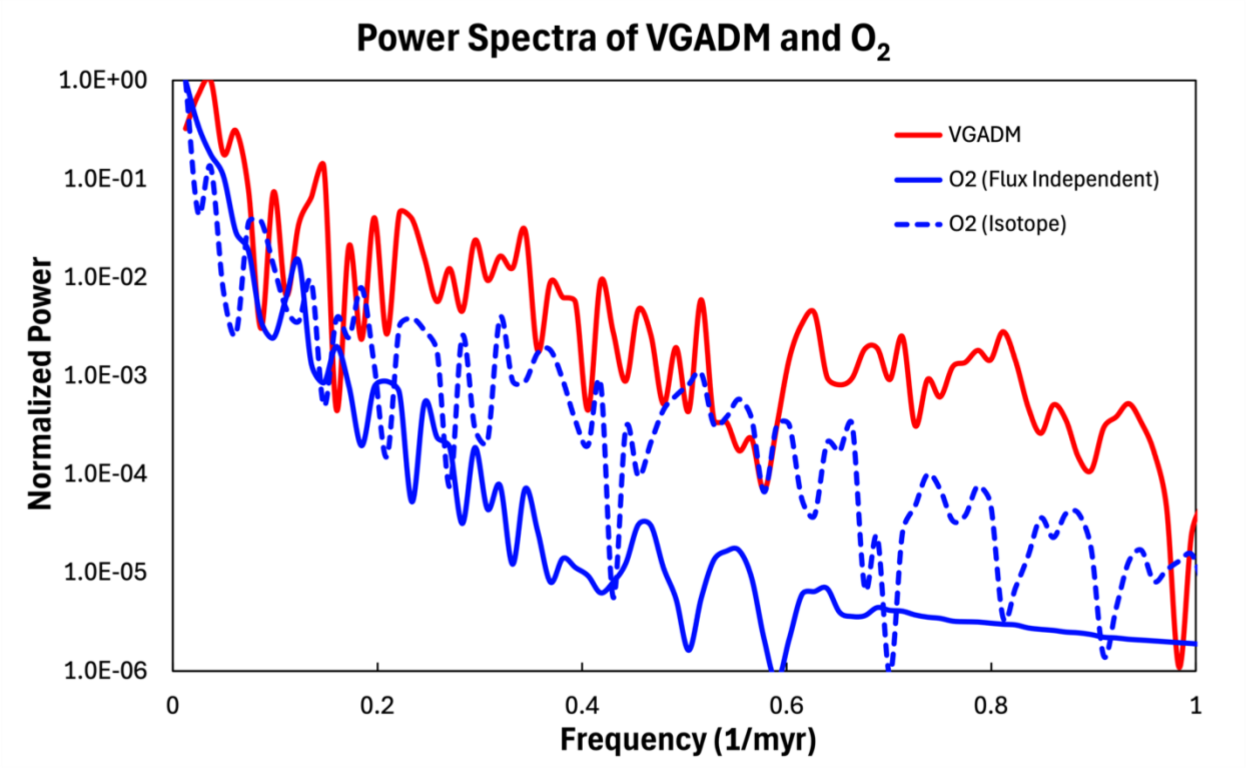

Figure S4. The power spectra of VGADM and O_2. Similar to Fig. 1B in the main text, but included that of the isotope O2 (blue dashed line), which possesses more power for frequencies higher than 0.2 (or the periods shorter than 30 myr).

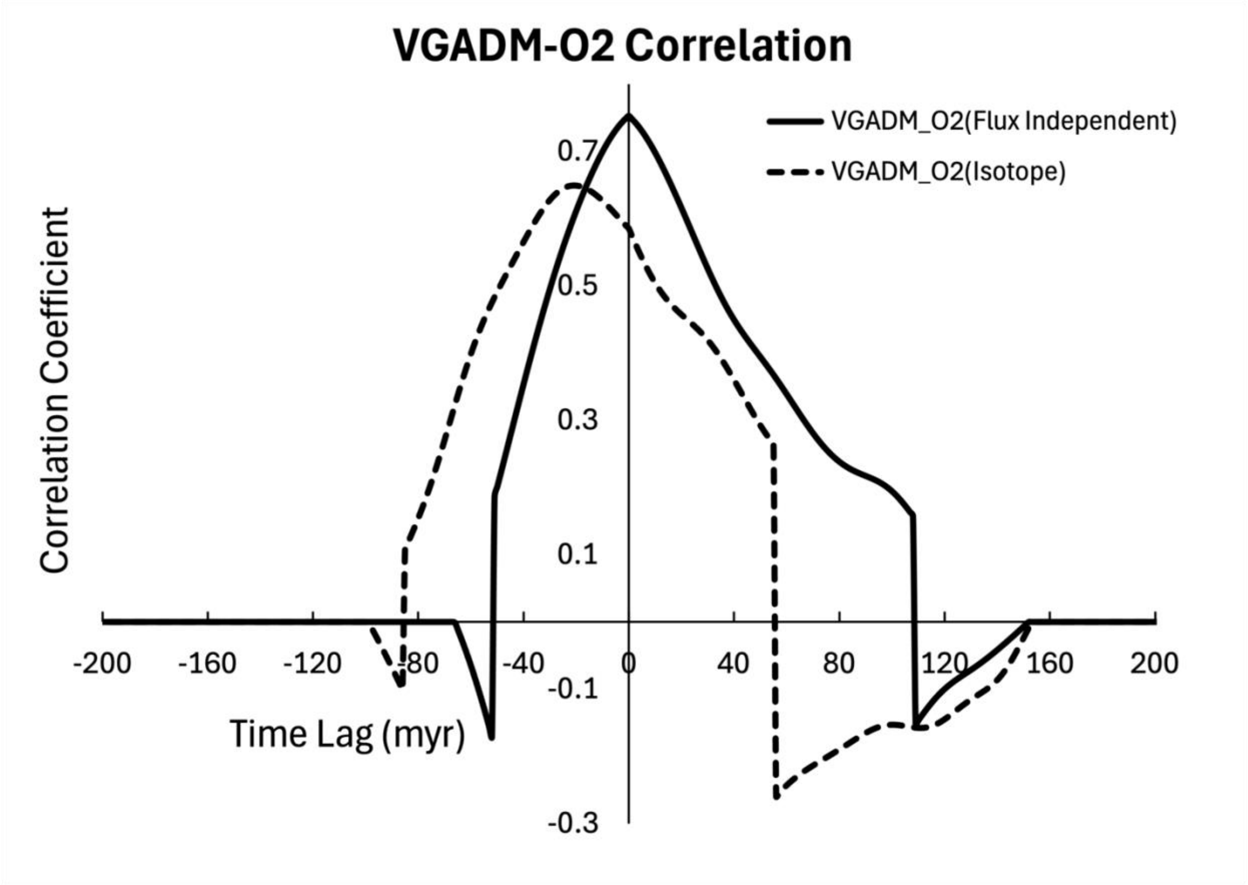

Figure S5. **The VGADM-O2 correlogram.** Here are the correlations between the flux independent O_2 and VGADM (the solid line), and the correlation between the isotope O_2 and VGADM (the dashed line). The data time series are filtered with the 20 myr high-frequency band pass. Both are highly correlated, but the maximum correlation occurs at a -25 myr time lag for the isotope O_2, but with no time-lag for the flux-independent O_2.

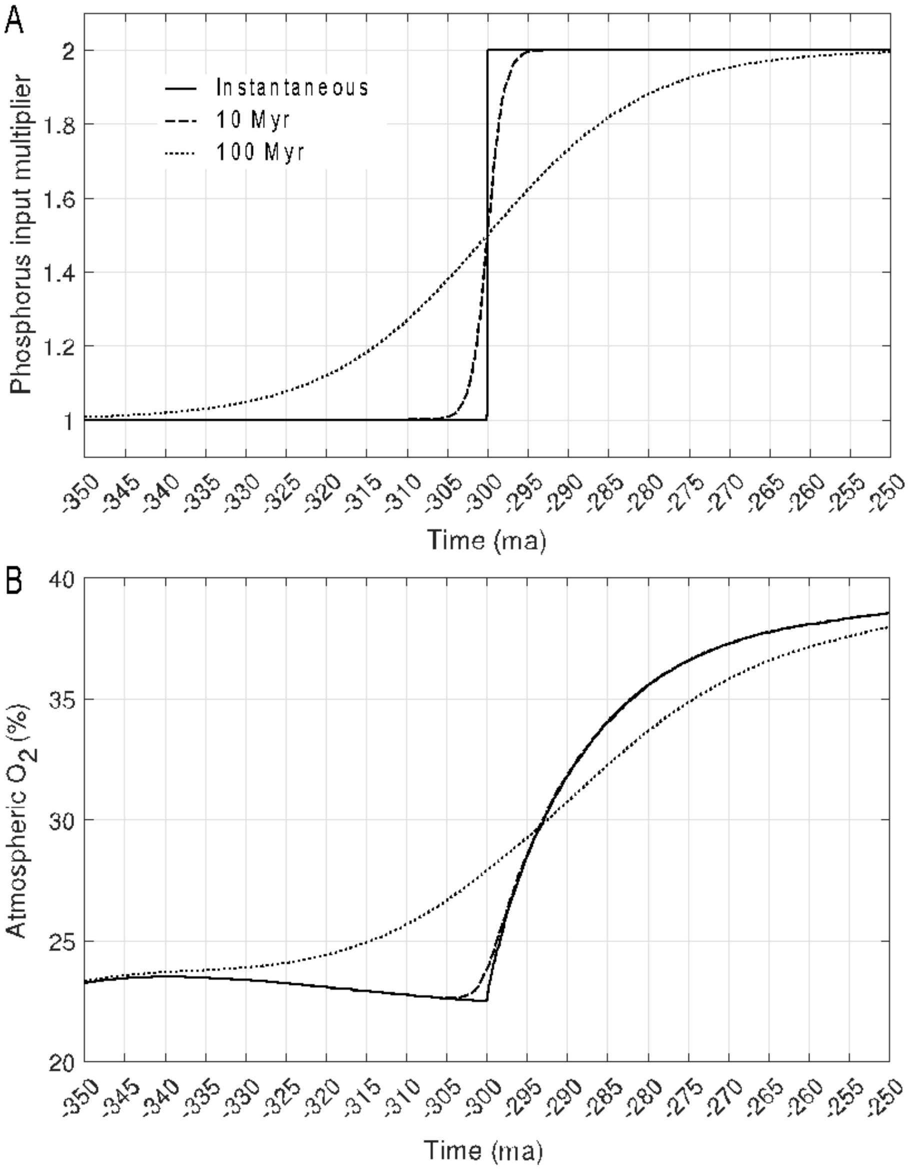

Figure S6. O_2 response lag to tectonic forcing. Examples of O_2 response lags in the SCION global biogeochemical model v1.2 (https://github.com/bjwmills/SCION; https://doi.org/10.1016/j.gloplacha.2025.104791). (A) Input forcing on different timescales, represented here by an arbitrary increase in riverine phosphorus input to the oceans. (B) Model atmospheric O_2. Input lag is reduced as timeframe of change to model processes becomes longer, to the point at which the lag is within the time uncertainty on the proxy records for atmospheric O_2 when the input varies on a ~50 myr timeframe or more.

Data S1: The full VGADM time series for the correlation analysis. The time series of the VGADM mean (column 2), standard deviation (column 3) and mode (column 4) in the past 540 myr. The data are derived by applying the 2-myr bandpass filter to MCADMv1a (*31*), and are redistributed on the uniform age grid (column 1) with the 1-myr grid size. The unit of the VGADM data is 10^{23} Nm^3.

Data S2: The independent O_2 content time series for the correlation analysis. This is similar to Data S1, but includes the mid (column 2), min (column 3) and max (column 4) values of the independent O_2 on the same age grid (column 1). The unit of the O2 data is percent (%).

REFERENCES AND NOTES

1. M. R. Walter, R. Buick, J. S. R. Dunlop, Stromatolites 3,400–3,500 Myr old from the North Pole area, Western Australia. *Nature* **284**, 443–445 (1980).

2. E. A. Bell, P. Boehnke, T. M. Harrison, W. L. Mao, Potentially biogenic carbon preserved in a 4.1 billion-year-old zircon. *Proc. Natl. Acad. Sci. U.S.A.* **112**, 14518–14521 (2015).

3. M. S. Dodd, D. Papineau, T. Grenne, J. F. Slack, M. Rittner, F. Pirajno, J. O'Neil, C. T. S. Little, Evidence for early life in Earth's oldest hydrothermal vent precipitates. *Nature* **543**, 60–64 (2017).

4. J. A. Tarduno, R. D. Cottrell, W. J. Davis, F. Nimmo, R. K. Bono, A hadean to Paleoarchean geodynamo recorded by single zircon crystals. *Science* **349**,521–524 (2015).

5. J. A. Tarduno, R. D. Cottrell, R. K. Bono, H. Oda, W. J. Davis, M. Fayek, O. van't Erve, F. Nimmo, W. Huang, E. R. Thern, S. Fearn, G. Mitra, A. V. Smirnov, E. G. Blackman, Paleomagnetism indicates that primary magnetite in zircon records a strong Hadean geodynamo. *Proc. Natl. Acad. Sci. U.S.A.* **117**, 2309–2318 (2020).

6. D. A. Brain, F. Bagenal, Y.-J. Ma, H. Nilsson, G. Stenberg Wieser, Atmospheric escape from unmagnetized bodies. *J. Geophys. Res. Planets* **121**, 2364–2385 (2016).

7. V. S. Airapetian, A. Glocer, G. Gronoff, E. Hébrard, W. Danchi, Prebiotic chemistry and atmospheric warming of early Earth by an active young Sun. *Nat. Geosci.* **9**, 452–455 (2016).

8. V. S. Airapetian, A. Glocer, G. V. Khazanov, R. O. P. Loyd, K. France, J. Sojka, W. C. Danchi, M. W. Liemohn, How hospitable are space weather affected habitable zones? The role of ion escape. *Astrophys. J. Lett.* **836**, L3 (2017).

9. V. S. Airapetian, R. Barnes, O. Cohen, G. A. Collinson, W. C. Danchi, C. F. Dong, A. D. Del Genio, K. France, K. Garcia-Sage, A. Glocer, N. Gopalswamy, J. L. Grenfell, G. Gronoff, M. Güdel, K. Herbst, W. G. Henning, C. H. Jackman, M. Jin, C. P. Johnstone, L. Kaltenegger, C. D. Kay, K. Kobayashi, W. Kuang, G. Li, B. J. Lynch, T. Lüftinger, J. G. Luhmann, H. Maehara, M.

G. Mlynczak, Y. Notsu, R. A. Osten, R. M. Ramirez, S. Rugheimer, M. Scheucher, J. E. Schlieder, K. Shibata, C. Sousa-Silva, V. Stamenković, R. J. Strangeway, A. V. Usmanov, P. Vergados, O. P. Verkhoglyadova, A. A. Vidotto, M. Voytek, M. J. Way, G. P. Zank, Y. Yamashiki, Impact of space weather on climate and habitability of terrestrial-type exoplanets. *Int. J. Astrobiol.* **19**, 136–194 (2020).

10. J. Varela, A. S. Brun, A. Strugarek, V. Réville, P. Zarka, F. Pantellini, On Earth's habitability over the Sun's main-sequence history: Joint influence of space weather and Earth's magnetic field evolution. *Mon. Not. R. Astron. Soc.* **525**, 4008–4025 (2023).

11. M. Lingam, Revisiting the biological ramifications of variations in Earth's magnetic field. *Asptrophys. J. Lett.* **874**, L28 (2019).

12. R. Ramstad, S. Barabash, Do intrinsic magnetic fields protect planetary atmospheres from stellar winds? Lessons from ion measurements at Mars, Venus, and Earth. *Space Sci. Rev.* **217**, 36 (2021).

13. A. S. Atkinson, D. Alexander, A. O. Farrish, Exploring the effects of stellar magnetism on the potential habitability of exoplanets. *Astrophys. J.* **969**, 147 (2024).

14. W. Huang, J. A. Tarduno, T. Zhou, M. Ibañez-Mejia, L. D. Olmo-Barbosa, E. Koester, E. G. Blackman, A. V. Smirnov, G. Ahrendt, R. D. Cottrell, K. P. Kodama, R. K. Bono, D. G. Sibeck, Y.-X. Li, F. Nimmo, S. Xiao, M. K. Watkeys, Near-collapse of the geomagnetic field may have contributed to atmospheric oxygenation and animal radiation in the Ediacaran Period. *Commun. Earth Environ.* **5**, 207 (2024).

15. I. H. Campbell, C. M. Allen, Formation of supercontinents linked to increases in atmospheric oxygen. *Nat. Geosci.* **1**, 554–558 (2008).

16. H. D. Holland, Volcanic gases, black smokers, and the great oxidation event. *Geochim. Cosmochim. Acta* **66**, 3811–3826 (2002).

17. J. Dannberg, R. Gassmöller, D. Thallner, F. LaCombe, C. Sprain, Changes in core-mantle boundary heat flux patterns throughout the supercontinent cycle. *Geophys. J. Int.* **237**, 1251–1274 (2024).

18. M. Korte, C. G. Constable, C. J. Davies, S. Panovska, Indicators of Mantle Control on the geodynamo from observations and simulations. *Front. Earth Sci.* **10**, 957815 (2022).

19. J. Farquhar, H. Bao, M. Thiemens, Atmospheric influence of Earth's earliest sulfur cycle. *Science* **289**, 756–758 (2000).

20. T. W. Lyons, C. W. Diamond, N. J. Planavsky, C. T. Reinhard, C. Li, Oxygenation, life, and the planetary system during Earth's middle history: An overview. *Astrobiology* **21**, 906–923 (2021).

21. B. J. W. Mills, A. J. Krause, I. Jarvis, B. D. Cramer, Evolution of atmospheric O_2 through the Phanerozoic, revisited. *Annu. Rev. Earth Planet. Sci.* **51**, 253–276 (2023).

22. A. Bekker, H. D. Holland, P. L. Wang, D. Rumble III, H. J. Stein, J. L. Hannah, L. L. Coetzee, N. J. Beukes, Dating the rise of atmospheric oxygen. *Nature* **427**, 117–120 (2004).

23. D. C. Catling, *The Great Oxidation Event Transition. Treatise on Geochemistry* (Elsevier, ed. 2, 2014), pp. 191–233.

24. J. Krissansen-Totton, M. A. Kipp, D. C. Catling, Carbon cycle inverse modeling suggests large changes in fractional organic burial are consistent with the carbon isotope record and may have contributed to the rise of oxygen. *Geobiology* **19**, 342–363 (2021).

25. L. M. Och, G. A. Shields-Zhou, The neoproterozoic oxygenation event: Environmental perturbations and biogeochemical cycling. *Earth Sci. Rev.* **110**, 26–57 (2012).

26. J. Larmor, How could a rotating body such as the Sun become a magnet?. *Rep. Br. Ass. Advmt. Sci.* **87**, 159–160 (1919).

27. S. I. Braginsky, P. H. Roberts, Equations governing convection in Earth's core and the geodynamo. *Geophys. Astrophys. Fluid Dyn.* **79**, 1–97 (1995).

28. G. A. Glatzmaier, P. H. Roberts, A three-dimensional self-consistent computer simulation of a geomagnetic field reversal. *Nature* **377**, 203–209 (1995).

29. P. E. Driscoll, C. Wilson, Paleomagnetic biases inferred from numerical dynamos and the search for geodynamo evolution. *Front. Earth Sci.* **6**, 113 (2018).

30. J. E. T. Channel, B. S. Singer, B. R. Jicha, Timing of Quaternary geomagnetic reversals and excursions in volcanic and sedimentary archives. *Quat. Sci. Rev.* **228**, 106114 (2020).

31. R. K. Bono, G. A. Paterson, A. J. Biggin, MCADAM: A continuous paleomagnetic dipole moment model for at least 3.7 billion years. *Geophys. Res. Lett.* **49**, e2022GL100898 (2022).

32. C. Kato, Y. Usui, M. Sato, A brief review of single silicate crystal paleointensity: Rock-magnetic characteristics, mineralogical backgrounds, methods and applications. *Earth Planets Space* **76**, 49 (2024).

33. W. K. Peterson, D. A. Brain, N. R. Schnepf, Y. Dong, P. Chamberlin, A. W. Yau, Atmospheric escape from Earth and Mars: Response to solar and solar wind drivers of oxygen escape. *Geophys. Res. Lett.* **51**, e2023GL107675 (2024).

34. K. Seki, R. C. Elphic, M. Hirahara, T. Terasawa, T. Mukai, On atmospheric loss of oxygen ions from Earth through magnetospheric processes. *Science* **291**, 1939–1941 (2001).

35. T. M. Lenton, S. J. Daines, B. J. W. Mills, COPSE reloaded: An improved model of biogeochemical cycling over Phanerozoic time. *Earth Sci. Rev.* **178**, 1–28 (2018).

36. M. Persson, Y. Futaana, R. Ramstad, K. Masunaga,H. Nilsson, M. Hamrin, A. Federov, S. Barabash, The Venusian atmospheric oxygen ion escape: Extrapolation to the early solar system. *J. Geophys. Res. Planets* **125**, e2019JE006336 (2020).

37. T. Zhou, J. A. Tarduno, F. Nimmo, R. D. Cottrell, R. K. Bono, M. Ibanez-Mejia, W. Huang, M. Hamilton, K. Kodama, A. V. Smirnov, B. Crummins, F. Padgett III, Early Cambrian renewal of the geodynamo and the origin of inner core structure. *Nat. Commun.* **13**, 4161 (2022).

38. N. Zhang, S. Zhong, Heat fluxes at the Earth's surface and core-mantle boundary since Pangea formation and their implications for the geomagnetic superchrons. *Earth Planet. Sci. Lett.* **306**, 205–216 (2011).

39. U. R. Christensen, Geodynamo models with a stable layer and heterogeneous heat flow at the top of the core. *Geophys. J. Int.* **215**, 1338–1351 (2018).

40. L. J. Alcott, C. Walton, N. J. Planavsky, O. Shorttle, B. J. W. Mills, Crustal carbonate build-up as a driver for Earth's oxygenation. *Nat. Geosci.* **17**, 458–464 (2024).

41. J. J. Williams, B. J. W. Mills, T. M. Lenton, A tectonically driven Ediacaran oxygenation event. *Nat. Commun.* **10**, 2690 (2019).

42. J. Eguchi, C. W. Diamond, T. W. Lyons, Proterozoic supercontinent break-up as a driver for oxygenation events and subsequent carbon isotope excursions. *PNAS Nexus* **1**, 1–10 (2022).

43. I. J. Glasspool, A. C. Scott, D. Waltham, N. Pronina, L. Shao, The impact of fire on the late Paleozoic Earth system. *Front. Plant Sci.* **6**, 756 (2015).

44. R. A. Berner, D. E. Canfield, A new model for atmospheric oxygen over Phanerozoic time. *Am. J. Sci.* **289**, 333–361 (1989).

45. C. T. Edwards, M. R. Saltzman, D. L. Royer, D. A. Fike, Oxygenation as a driver of the great ordovician biodiversification event. *Nat. Geosci.* **10**, 925–929 (2017).

46. D. E. Canfield, A new model for Proterozoic ocean chemistry. *Nature* **396**, 450–453 (1998).

47. E. A. Sperling, C. J. Wolock, A. S. Morgan, B. C. Gill, M. Kunzmann, G. P. Halverson, F. A. Macdonald, A. H. Knoll, D. T. Johnston, Statistical analysis of iron geochemical data suggests limited late Proterozoic oxygenation. *Nature* **523**, 451–4 (2015).

48. A. J. Krause, B. J. W. Mills, S. Zhang, N. J. Planavsky, T. M. Lenton, S. W. Poulton, Stepwise oxygenation of the Paleozoic atmosphere. *Nat. Commun.* **9**, 4081 (2018).

49. C. Chatfield, *The Analysis of Time Series: An Introduction* (Chapman & Hall, ed. 5, 1996).

50. D. Surcel, R. Larprise, A general filter for stretched-grid models: Application in Cartesian geometry. *Mon. Weather Rev.* **139**, 1637–1653 (2011)

51. A. R. Brenner, R. R. Fu, A. R. C. Kylander-Clark, B. J. Foley, Plate motion and a dipolar geomagnetic field at 3.25 Ga. *Proc. Natl. Acad. Sci. U.S.A.* **119**, e2210258119 (2022).
